# Supplementary material for: A quantitative inventory of yeast P body proteins reveals principles of composition and specificity
Source: eLife. 2020 Jun 19;9:e56525. doi: 10.7554/eLife.56525 (PMC7373430; doi:10.7554/eLife.56525)
Supplement: Supplementary file 2. [file elife-56525-supp2.docx]

| Proteins | Partition  Coefficient | P body concentrations (uM) | Cytoplasm concentrations (uM) | Exchange rate  (s^-1^)^**^ | Fractional recovery |
| --- | --- | --- | --- | --- | --- |
| Dcp2 | 133 ± 13 | 15 ± 1 | 0.12 ± 0.01 | ~ 0.0003^***^ | 0 |
| Edc3 | 133 ± 8 | 12 ± 1 | 0.09 ± 0.01 | ~ 0.0005^***^ | 0 |
| Pat1 | 107 ± 12 | 12 ± 1 | 0.12 ± 0.01 | 0.009 ± 0.002 | 0.52 ± 0.06 |
| Xrn1 | 53 ± 3 | 11 ± 1 | 0.22 ± 0.01 | 0.028 ± 0.002 | 0.74 ± 0.04 |
| Lsm1 | 52 ± 4 | 8.9 ± 0.6 | 0.17 ± 0.01 | 0.022 ± 0.002 | 0.67 ± 0.03 |
| Upf1 | 39 ± 3 | 5.1 ± 0.4 | 0.13 ± 0.01 | 0.011 ± 0.002 | 0.47 ± 0.03 |
| Dhh1 | 30 ± 2 | 10 ± 1 | 0.38 ± 0.02 | 0.035 ± 0.003 | 0.75 ± 0.04 |
| Not2 | 14 ± 1 | 1.7 ± 0.2 | 0.13 ± 0.01 | 0.036 ± 0.003 | 0.82 ± 0.05 |
| CCR4 | 13 ± 1 | 2.0 ± 0.2 | 0.18 ± 0.01 | 0.026 ± 0.003 | 0.84 ± 0.05 |
| Pop2 | 12 ± 1 | 2.6 ± 0.2 | 0.22 ± 0.01 | 0.026 ± 0.002 | 0.85 ± 0.04 |
| Upf2 | 12 ± 1 | 1.1 ± 0.1 | 0.09 ± 0.01 | 0.11 ± 0.01 | 0.92 ± 0.06 |
| Upf3 | 9.6 ± 0.8 | 1.4 ± 0.1 | 0.15 ± 0.01 | 0.064 ± 0.007 | 0.91 ± 0.05 |
| Hek2 | 5.9 ± 0.8 | 1.0 ± 0.1 | 0.16 ± 0.01 | 0.091 ± 0.008 | 0.99 ± 0.05 |
| Eap1 | 5.8 ± 0.5 | 1.2 ± 0.1 | 0.22 ± 0.01 | 0.072 ± 0.006 | 1.00 ± 0.06 |
| Edc1 | 5.7 ± 0.5 | 0.8 ± 0.1 | 0.15 ± 0.01 | 0.23 ± 0.03 | 1.00 ± 0.07 |
| Bre5 | 5.6 ± 0.4 | 1.5 ± 0.1 | 0.28 ± 0.01 | 0.11 ± 0.01 | 0.97 ± 0.06 |
| Psp2 | 4.8 ± 0.5 | 0.7 ± 0.1 | 0.15 ± 0.01 | 0.13 ± 0.01 | 1.00 ± 0.07 |
| Sbp1 | 4.5 ± 0.4 | 4.6 ± 0.4 | 1.1 ± 0.05 | 0.069 ± 0.007 | 0.94 ± 0.05 |
| Ssd1 | 4.5 ± 0.4 | 1.1 ± 0.1 | 0.25 ± 0.02 | 0.052 ± 0.008 | 0.92 ± 0.06 |

**Supplementary File 2. Dynamics and partitioning of P body proteins^*^**

* Mean ± SEM for partitioning coefficient, P body concentrations, Cytoplasm concentrations, Exchange rate and Fractional recovery.

** Exchange rate is exponential rate constant of exchange (k).

*** FRAP curves of Dcp2 and Edc3 are almost flat that the software can only fit ambiguous values.
